# A novel sympathetic neuronal GABAergic signalling system regulates NE release to prevent ventricular arrhythmias after acute myocardial infarction

**DOI:** 10.1111/apha.13315

**Published:** 2019-06-12

**Authors:** Yugen Shi, Yan Li, Jie Yin, Hesheng Hu, Mei Xue, Xiaolu Li, Wenjuan Cheng, Ye Wang, Xinran Li, Yu Wang, Jiayu Tan, Suhua Yan

**Affiliations:** ^1^ Department of Cardiology Shandong Provincial Qianfoshan Hospital, Shandong University Shandong China; ^2^ Medical Research Center Shandong Provincial Qianfoshan Hospital, Shandong University Shandong China; ^3^ School of Medicine Shandong University Shandong China

**Keywords:** arrhythmias, GABA, myocardial infarction, superior cervical ganglia, sympathetic nerve activity

## Abstract

**Aim:**

Overactivation of the sympathetic nerve may lead to severe ventricular arrhythmias (VAs) after myocardial infarction (MI). Thus, targeting sympathetic nerve activity is an effective strategy to prevent VAs clinically. The superior cervical ganglion (SCG), the extracardiac sympathetic ganglion innervating cardiac muscles, has been found to have a GABAergic signalling system, the physiological significance of which is obscure. We aimed to explore the functional significance of SCG post MI and whether the GABAergic signal system is involved in the process.

**Methods:**

Adult male Sprague‐Dawley rats were divided into seven different groups. Rats in the MI groups underwent ligation of the left anterior descending coronary artery. All animals were used for electrophysiological testing, renal sympathetic nerve activity (RSNA) testing, and ELISA. Primary SCG sympathetic neurons were used for the in vitro study.

**Results:**

The GABA_A_ receptor agonist muscimol significantly decreased the ATP‐induced increase in intracellular Ca^2+^ (*P < *0.05). GABA treatment in MI rats significantly attenuated the level of serum and cardiac norepinephrine (NE; *P < *0.05). Sympathetic activity and inducible VAs were also lower in MI + GABA rats than in MI rats (*P < *0.05). Knockdown of the GABA_A_Rs β_2_ subunit (GABA_A_Rβ_2_) in the SCG of MI rats increased the NE levels in serum and cardiac tissue, RSNA and inducible VAs compared with vehicle shRNA (*P < *0.05).

**Conclusion:**

The GABAergic signalling system is functionally expressed in SCG sympathetic neurons, and activation of this system suppresses sympathetic activity, thereby facilitating cardiac protection and making it a potential target to alleviate VAs.

## 1 INTRODUCTION

Cardiac sympathetic nerve innervation plays important roles in cardiac physiologic and pathophysiologic processes, especially in ventricular arrhythmias (VAs) after acute myocardial infarction (MI). Sympathetic excitation because of cardiac and extracardiac sympathetic nerve remodelling directly leads to ventricular arrhythmogenesis.^1^, ^2^, ^3^, ^4^ Strategies to lower sympathetic nerve activities have been effective in reducing VAs by targeting cardiac and extracardiac sympathetic nerves. For example, some clinical approaches, such as thoracic epidural anaesthesia,^5^ sympathetic ganglia‐targeted therapy^6^ and renal artery denervation,^7^, ^8^ reportedly result in more than an 80% reduction in VAs in patients.^5^ However, the indications and techniques make it difficult for all VA patients to benefit from the above protocols, especially patients with a high risk of VAs after MI. Moreover, beta blockers are well used clinically, but systemic side effects, especially in severe atrioventricular block and the respiratory system, have restricted their usage. Therefore, more effective methods to lower sympathetic nerve activity must be explored. In this study, we reinvestigated the superior cervical ganglion (SCG), which innervates the myocardium,^9^ and revealed a new target for modulation of extracardiac sympathetic nerve activity to prevent VAs in MI rats.

The anatomic evidence that the SCG innervates the heart has long been reported,^9^, ^10^ but the exact physiological and pathophysiological roles of SCG postsynaptic neurons have barely been explained. Postganglionic neurons in the SCG are believed to be positively regulated by presynaptic acetylcholine (ACh) nerve fibres, while the other regulating mechanisms remain obscure even though several hypotheses have been raised.^11^, ^12^ For example, Wolff et al^11^ suggested that neurons in the SCG releasing γ‐aminobutyric acid (GABA) regulate nerve activity, while Diana Elinos et al^13^ supported a different degree of ACh and GABA segregation in presynaptic ACh fibres. The common finding in all reported articles is that the SCG expresses GABA, GABA receptors and GABA synthetic enzymes^14^ by which it may regulate ganglionic transmission^14^ and decrease blood pressure and heart rate (HR) physiologically.^15^ Nonetheless, the mechanisms require further investigation.

The GABAergic signalling system, which includes GABA, GABA receptors and GABA synthetic enzymes, has a well‐known inhibitory role in maintaining the balance of glutamic acid in the central nervous system (CNS).^16^ Moreover, GABA_A_ and GABA_B_ receptors participate in cardiac function modulation by tonic sympathetic outflow in the paraventricular nucleus of the hypothalamus^17^; exogenous GABA in the CNS also mediates autonomic nerve activity to decrease coronary vessel resistance and exert an anti‐arrhythmic effect on ischemic heart tissue.^18^ Outside the CNS, many studies have discovered new functions of this system in peripheral organs such as the pancreatic islet,^19^ liver^20^, ^21^ and human fallopian tube,^22^ as well as in the cardiovascular system. Injection of exogenous GABA via the caudal vein significantly decreases the incidence of arrhythmias induced by aconitine,^23^ indicating a potential peripheral therapeutic effect of the GABAergic signalling system in the treatment of cardiovascular diseases such as heart failure, MI and arrhythmias. Consistent with the above reports, we found that GABA_A_ receptors and GABA synthetic enzymes are expressed in SCG neurons. Thus, we hypothesized that the GABAergic signalling system in the SCG plays an important role in VAs after MI.

In addition, overexpression of P2X receptors (P2XRs) in the SCG might cause over activation of cardiac sympathetic nerves in MI animal models,^24^, ^25^, ^26^ and activation of P2XRs induces elevated intracellular Ca^2+ ^levels,^27^ indicating that the P2XRs/Ca^2+^ pathway underlies the mechanism by which SCG neurons modulate cardiac sympathetic activity after MI. Negative cross talk between GABA_A_ receptors and P2XRs has been reported for years outside the CNS,^28^ but their functional relationships must be determined.

## 2 RESULTS

### 2.1 GABAergic signalling system expression in SCG sympathetic neurons

**Figure 1:**
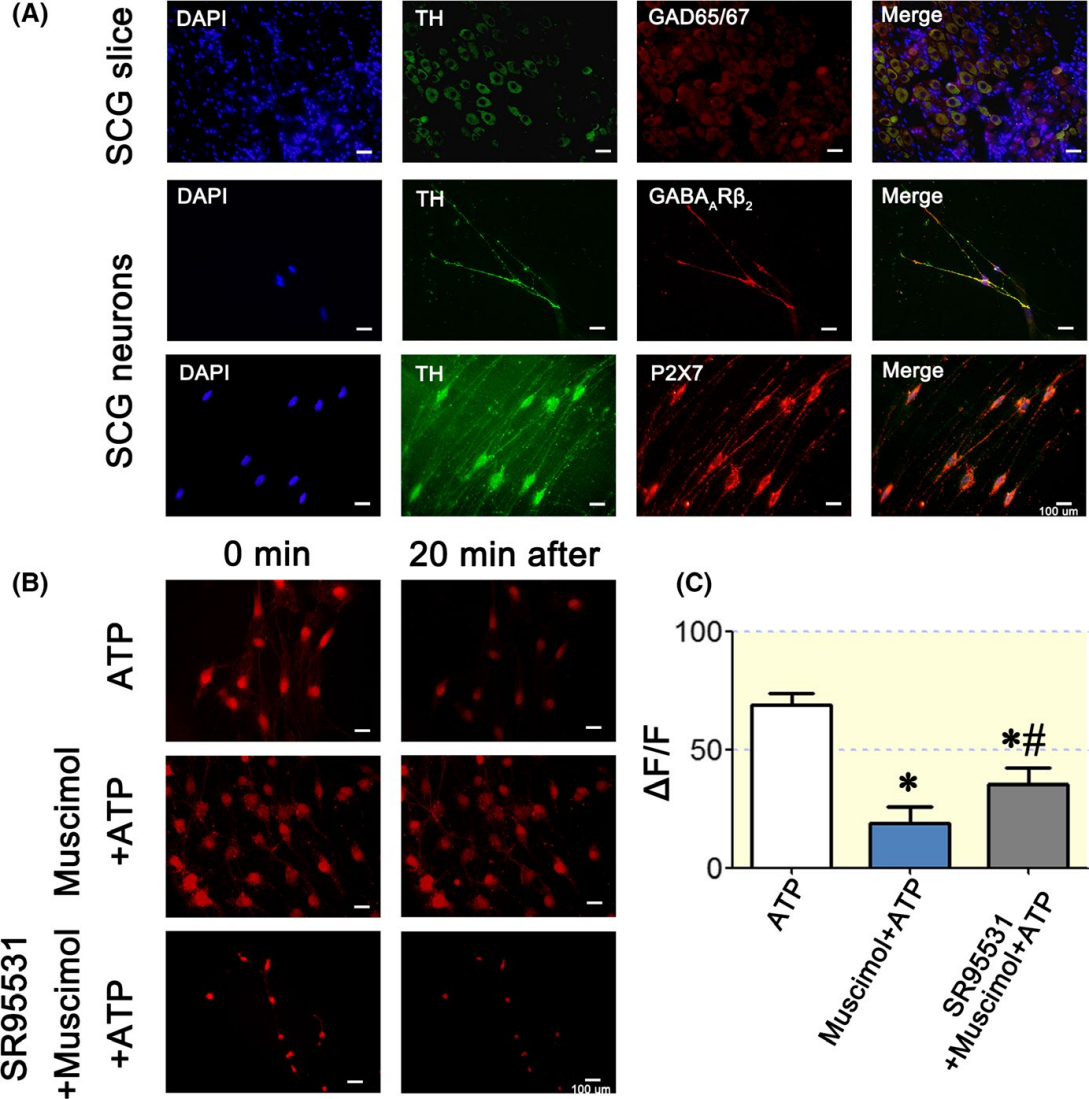
GABAergic signalling system and P2X7Rs expressed SCG neurons. A: TH‐positive (green) neurons in rat SCG slices were positively stained for the GABA synthetic enzyme GAD65/67 (red in the first panel). GABA_A_R subunits (GABA_A_Rβ_2_) were located on both the cell body and axons (red colour in the second panel) of the SCG TH‐positive neurons (green colour). The distribution pattern of the P2X7Rs (red colour in the third panel) is the same as that of GABA_A_Rβ_2_, which is located on TH‐positive neurons (green colour). B: Fura‐Red AM staining of cultured SCG neurons for testing the intracellular Ca^2+^ concentration ([Ca^2+^]i). A higher fluorescence intensity indicates a lower [Ca^2+^]i. The representative pictures show the 0‐ and 20‐minute results. ATP increased [Ca^2+^]i significantly, but muscimol blocked the increase in Ca^2+^. The GABA_A_R antagonist SR95531 blocked the inhibitory effects of GABA on the ATP‐induced [Ca^2+^]i elevation. C: Statistical analysis of [Ca^2+^]i after treatment with ATP (n = 3), ATP + muscimol (n = 4), or SR95531 + ATP+muscimol (n = 5). At 20 minutes, [Ca^2+^]i in the ATP + muscimol group was significantly lower than that in the ATP group (*P < *0.05), and [Ca^2+^]i in the SR95531 + ATP+muscimol group was significantly higher than that in the ATP + muscimol group but was still lower than that in the ATP group, indicating that SR95531 partially blocked GABA_A_ receptors. The scale bar in each graph of A is 100 μm. One‐way ANOVA was used for analysis of these data. “*” *P < *0.05 compared with the ATP group. “#” *P < *0.05 compared with the ATP + muscimol group

Double staining of rat SCG slices with antibodies against TH and GABA synthetic enzymes (GAD65/67) was detected in sympathetic neurons (Figure 1A). Positive GABA_A_Rβ_2_ and P2X7R staining was detected on cultured rat SCG sympathetic neurons in the cell body and axons (Figure 1A).

Intracellular Ca^2+^ in SCG neurons was detected by Fura‐Red AM, with a higher fluorescence intensity indicating a lower intracellular Ca^2+^ concentration. After 20 minutes of detection, ATP (10 µM), a P2X7R agonist, significantly increased the intracellular Ca^2+^ concentration (Supplementary Video: ATP), while exogenous muscimol (10 µM) inhibited the increase in intracellular Ca^2+^ (Supplementary Video: ATP + muscimol). The selective GABA_A_R antagonist SR95531 reversed the inhibitory effect of muscimol on intracellular Ca^2+^ elevation (Figure 1B,C, Supplementary Video: SR95531 + ATP + muscimol).

These results indicated that muscimol modulates sympathetic neuron intracellular Ca^2+^ levels by P2X7Rs, but the physiological or pathophysiological functions of the GABAergic signalling system in SCG sympathetic neurons remain unclear.

### 2.2 Expression level of the GABA_A_ receptor on SCGs from MI rats

**Figure 2:**
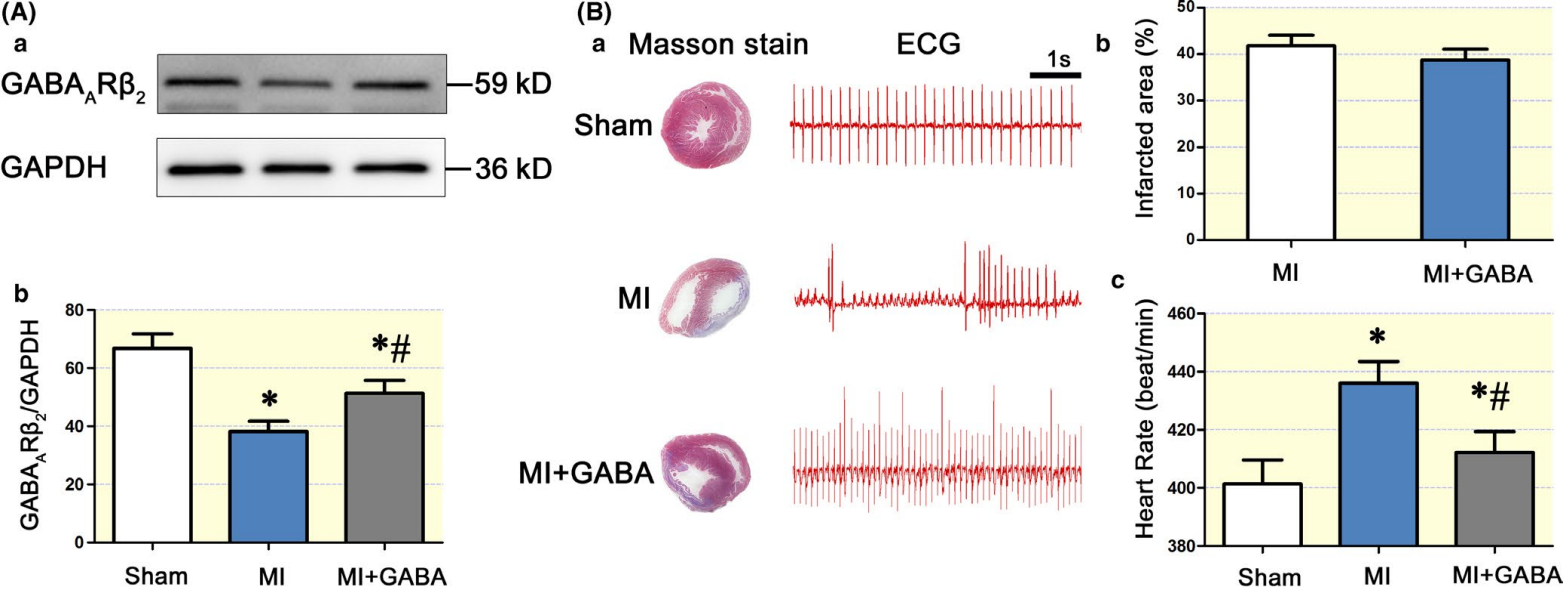
The SCG GABAergic signalling system was involved in MI. The protein band of GABA_A_Rβ_2_ was smaller in the MI rat SCG than in the sham surgery rat SCG, but the administration of GABA attenuated the change in GABA_A_Rβ_2 _levels (A). Compared with MI alone (n = 6), pretreatment with GABA (n = 4) before the MI procedure significantly reduced the rat HR (B, a and c), and the arrhythmias were relatively benign in the pretreatment group compared with those in the MI group. However, the infarct area of the heart in the MI and MI + GABA groups was not markedly different (B, a and b). “*” *P < *0.05 compared with the sham surgery group. “#” *P < *0.05 compared with the MI group. One‐way ANOVA was used to compare the different groups

To investigate the physiological and/or pathophysiological roles of the GABAergic signalling system in SCG sympathetic neurons, we tested the expression level of GABA_A_Rβ_2_ in SCGs from acute MI rats. MI rats exhibited lower SCG GABA_A_Rs levels than sham surgery rats, but oral administration of exogenous GABA improved GABA_A_ receptor expression (Figure 2A). Masson staining showed that GABA treatment resulted in a slightly smaller infarction area (Figure 2B, a and b, *P > *0.05) and significantly decreased the HR compared to MI alone (Figure 2B, c).

### 2.3 GABA inhibits sympathetic activity and improves cardiac function in MI rats

**Figure 3:**
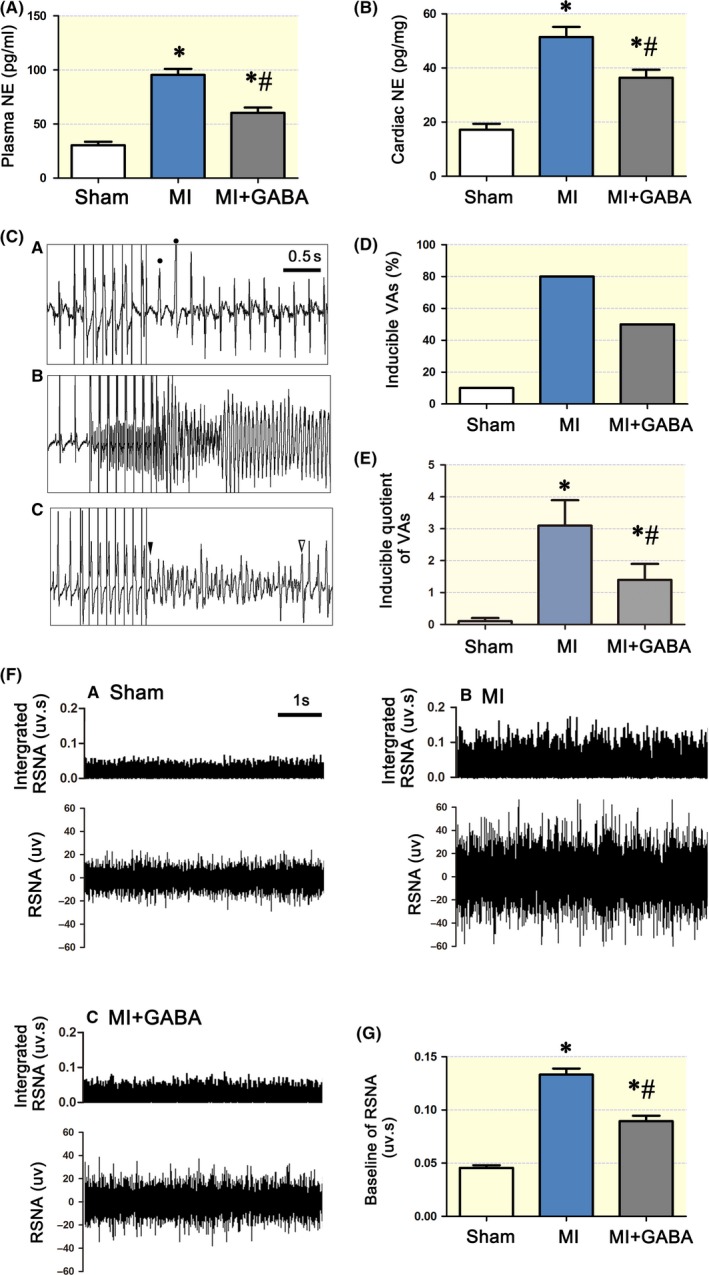
Exogenous GABA reduced peripheral sympathetic activity and the incidence of VAs. In MI rats, the plasma and cardiac NE levels were significantly higher than those in sham surgery rats (*, *P < *0.05, A and B). GABA reduced both plasma and cardiac NE levels in the MI + GABA group compared with MI alone (#, *P < *0.05, A and B). However, NE levels in the MI + GABA group were still higher than those in the sham surgery group (*, *P < *0.05, A and B). C, Recordings of typical inducible VAs with premature ventricular beats (PVC) (a), sustained ventricular tachycardia (b) and sustained ventricular fibrillation (c). D, The percentage of inducible total VAs/stimulation times was 1.5 times lower in the MI + GABA group than in the MI group (#, *P < *0.05), but those in both the MI and MI + GABA groups were higher than those in the sham surgery group (*, *P < *0.05). E, Statistical analysis of inducible VAs in the three groups. The inducible quotient of VAs was 3‐4 times higher in the MI group than in the sham surgery group (*, *P < *0.05) and was 2 times lower in the MI + GABA group than in the MI group (#, *P < *0.05). However, the inducible quotient of VAs in the MI + GABA group was still 2 times higher than that in the sham surgery group (*, *P < *0.05). F, Typical left RSNA and integrated RSNA recordings from an individual rat: a, sham surgery group; b, MI group; c: MI + GABA group. G, Statistical analysis of baseline RSNA in the 3 groups; “*” *P* < 0.05 compared with the sham surgery group and “#” *P* < 0.05 compared with the MI group; “•” indicates the PVC. “▼” marks the start of VF and “▼” is end of VF in this trace. NE, norepinephrine; VAs, ventricular arrhythmias; MI, myocardial infarction; RSNA, renal sympathetic nerve activity; PVC, premature ventricular beats. One‐way ANOVA was used to compare the different groups

To investigate peripheral sympathetic function, we measured the myocardial and plasma NE levels and RSNA. Plasma NE (Figure 3A) and myocardial NE (Figure 3B) levels were significantly upregulated by 3.76‐ and 3.04‐fold in the infarcted borderline area in the MI group compared with those in the sham surgery group. The exogenous GABA‐treated group exhibited significantly lower plasma and myocardial NE levels than the MI group.

To further elucidate the physiological effect of attenuated sympathetic reinnervation, we performed an electrophysiological study. Ventricular tachyarrhythmias were induced by programmed stimulation in infarcted rats, as shown in Figure 3C (a‐c). The arrhythmia score in the sham surgery group was very low. However, the arrhythmia score in the MI group was significantly higher than that in the sham surgery group (3.1 ± 0.8 vs 0.1 ± 0.1 *P < *0.05). In contrast, exogenous GABA significantly decreased the inducibility of ventricular tachyarrhythmia (Figure 3D,E). Therefore, exogenous GABA stabilized cardiac electrical activity and reduced the incidence of VAs.

As shown in Figure 3F‐G, the baseline RSNA of the MI group was significantly higher than that of the sham surgery group (*P < *0.05). Additionally, compared with the baseline RSNA of the MI group, the baseline RSNA of the MI group given exogenous GABA was significantly lower (0.017 ± 0.04 *P < *0.05).

In protocol 1, HR was higher in the MI group than in the sham surgery group (*P < *0.05). Compared to the MI group, the MI + GABA group had a lower HR (*P < *0.05). Hemodynamic analyses were performed to assess the influence of exogenous GABA on LV function 7 days post MI. The +dP/dt, −dP/dt and EF in the sham surgery group were significantly higher than those in the MI and MI + GABA groups (*P < *0.05). In the GABA‐treated MI group, cardiac function was significantly better than that in the MI group (Table 1) (*P < *0.05).

**Table 1:** Hemodynamic data based on pressure‐volume relations 7 days after MI

	Sham surgery	MI	MI + GABA
No. of rats	8	8	8
Body weight, g	254 ± 8.0	263 ± 8.6	264 ± 7.3
Heart rate, bpm	412 ± 9	429 ± 7*	420 ± 8#
LVESP (mm Hg)	107.2 ± 4.18	94.1 ± 3.9	97.8 ± 4.6
LVEDP (mm Hg)	5.7 ± 1.9	15.3 ± 3.7	12.7 ± 3.7
+dp/dtmax (mm Hg/s)	7238 ± 257	2973 ± 270*	5175 ± 273* #
−dp/dtmin (mm Hg/s)	6028 ± 293	2347 ± 214*	4114 ± 269* #
EF (%)	67.9 ± 4.2	35.4 ± 3.6*	44.7 ± 4.6* #

Note

Values are means ± SD.

Abbreviations: dP/dtmax and dP/dtmin, maximal slope of the systolic pressure increment and the diastolic pressure decrement; EF, left ventricular ejection fraction; LVEDP, left ventricular end‐diastolic pressure; LVESP, left ventricular end‐systolic pressure.

* *P* < 0.05 compared with the sham surgery group;

^#^ *P* < 0.05 compared with the MI group.

### 2.4 SCG GABA_A_Rβ_2_ knockdown increases RSNA and rat HR

**Figure 4:**
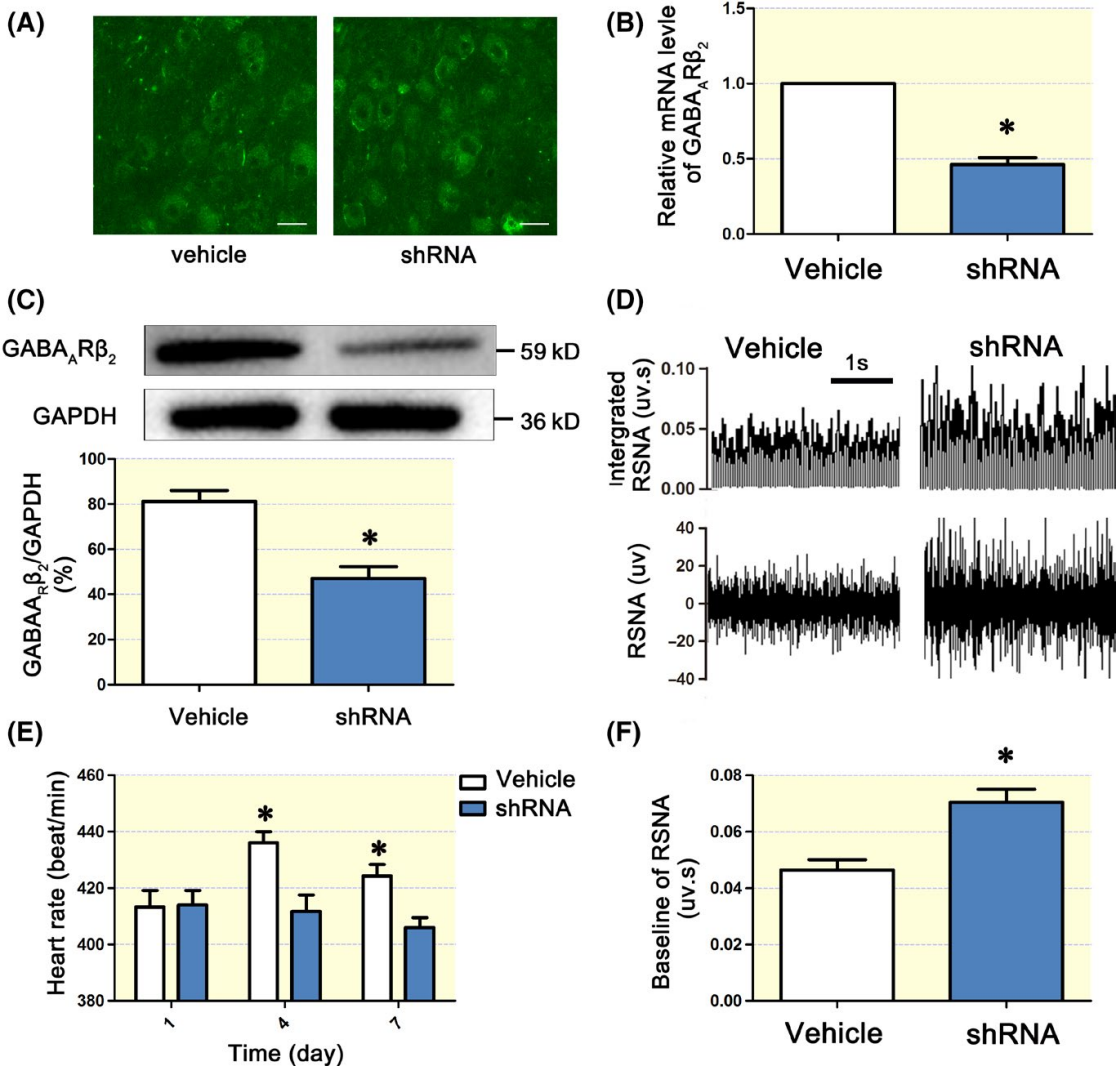
Knockdown of SCG GABA_A_Rβ_2_ increased RSNA and HR. Transduction efficiency after targeted injection of GABA_A_Rβ_2_ shRNA in the SCG was tested by qPCR and Western blotting. A, SCG slices showed a positive intracellular infection after 3 days of treatment with vehicle and shRNA vectors (both were GFP‐tagged). B, and C, GABA_A_Rβ_2_ was downregulated at both the protein and mRNA levels. D, Typical recordings of the RSNA in the vehicle and shRNA groups. Baseline RSNA was significantly increased in the GABA_A_Rβ_2_ shRNA group (*, *P < *0.05). F, Heart rate (HR) was significantly higher in the GABA_A_Rβ_2_ shRNA group than in the vehicle group on day 3 (*, *P < *0.05, E). The HR remained at a high level until day 7. RSNA, renal sympathetic nerve activity. SCG, superior cervical ganglion. The scale bar in each graph of A is 160 μm. “*” *P* < 0.05 compared with the vehicle group. One‐way ANOVA was used to compare the different groups

Four days after targeted microinjection of the GABA_A_Rβ_2_ shRNA lentiviral vector into the SCG, we tested the knockdown efficacy of the shRNAs. The mRNA and protein levels of GABA_A_Rβ_2_ in the SCG were significantly lower in the shRNA group than in the vehicle group (Figure 4B,C). In addition, compared with the vehicle, GABA_A_Rβ_2_ knockdown induced higher RSNA (Figure 4D,F). Rat HRs in the shRNA group were significantly increased from day 4 (Figure 4E).

### 2.5 Targeted knockdown of GABA_A_Rβ_2_ deteriorates NE levels, arrhythmia susceptibility and RSNA in MI rats

**Figure 5:**
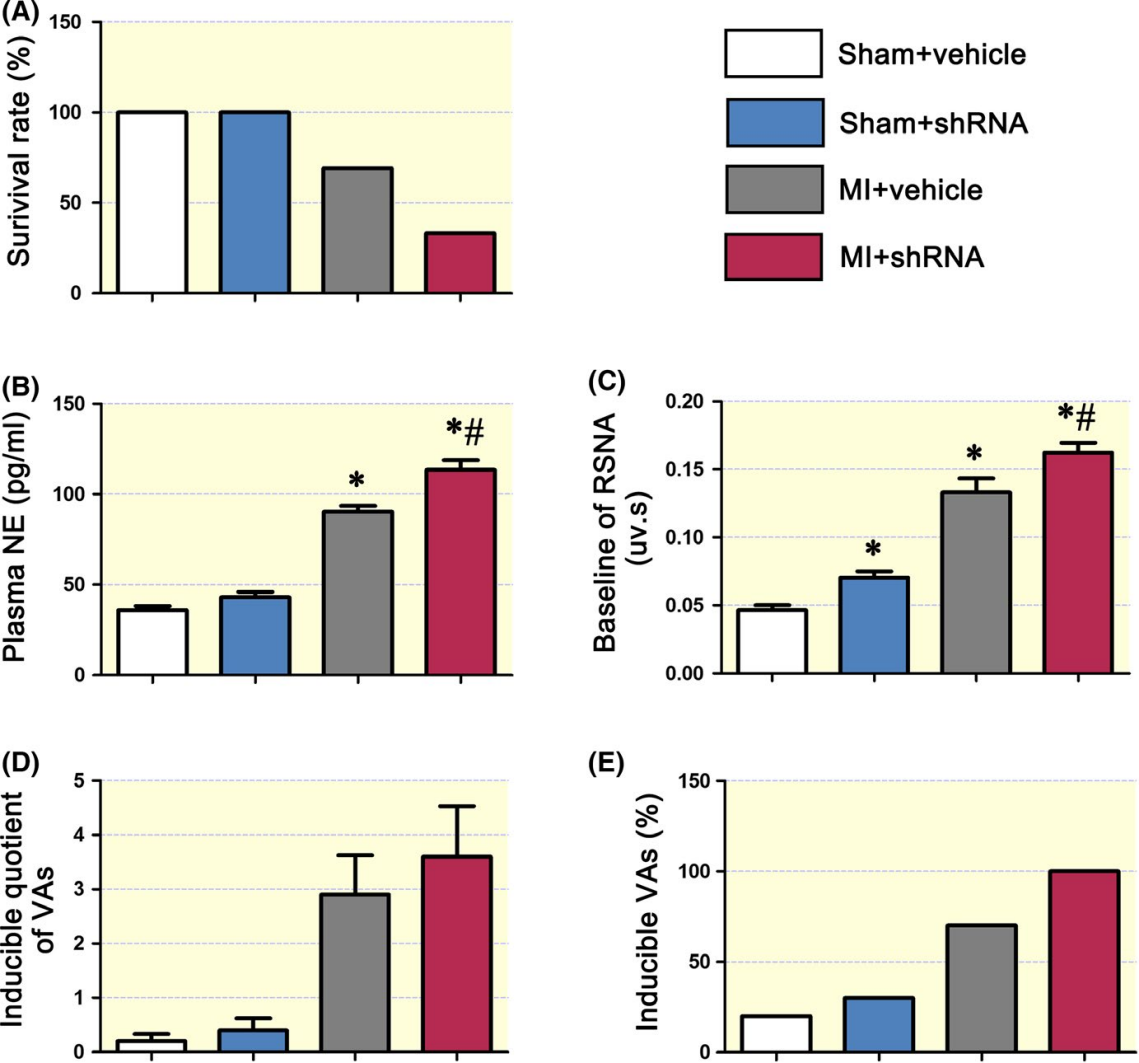
SCG GABA_A_Rβ_2_ knockdown induced sympathoexcitoxicity in MI rats. A, The survival rate of the SCG GABA_A_Rβ_2_ knockdown (shRNA) group was only 30%, and the survival rate of the MI group was approximately 70% at 7 days post MI. SCG‐targeted injection of vehicle and shRNA did not affect the survival rate. B, Plasma NE was higher in the MI + vehicle and MI + shRNA groups than in the control + vehicle group, and NE levels were highest in the MI + shRNA group. C, Baseline RSNA was higher in the shRNA only, MI + vehicle and MI + shRNA groups than in the control + vehicle group, and baseline RSNA was highest in the MI + shRNA group. D and E, The inducible quotient of ventricular arrhythmias (VAs) and inducible VAs were both higher in the MI groups (n = 6). The MI + shRNA group (n = 4) showed an even higher inducible quotient of VAs and inducible VAs than the MI + vehicle group. “*,” *P < *0.05 compared with the control + vehicle group; “#,” *P < *0.05 compared with the MI + vehicle group. Nonparametric testing was used to compare the different groups

Four days after targeted microinjection, rats underwent MI surgery. The survival rate of infarcted rats was lower in the MI + shRNA group than in the MI + vehicle group (33.3% vs 69.2%) seven days post MI (Figure 5A). Among these rats, five rats in the MI + shRNA died immediately after surgery because of fatal arrhythmias, and two rats died in the MI + vehicle group. Seven days after surgery, peripheral sympathetic function was investigated. Plasma NE levels and RSNA were higher in the MI + shRNA group than in the MI + vehicle group (*P* < 0.05; Figure 5B,C). Arrhythmia susceptibility was elevated in the MI + shRNA group, but there was no significant difference between the MI groups because of the lower survival rate in the MI + shRNA group (Figure 5D,E). Specifically, a lower survival rate and incidence of fatal arrhythmias post MI demonstrated that targeted knockdown of GABA_A_Rβ_2_ in the SCG deteriorated NE levels, RSNA and mortality rates after MI. Additionally, the expression level of GABA_A_Rβ_2_ in MI + shRNA rats was lower than that in the MI + vehicle group (Supplementary Figure 1).

## 3 DISCUSSION

### 3.1 GABA inhibition in the rat SCG

Early in 1986, Wolff, JR reported GABA‐like immunoreactive neurons in the rat SCG^29^ and considered this subpopulation of cells to be GABAergic interneurons with metabolic and morphogenetic activities in the rat SCG.^30^ Other reports have also discussed tonic GABA inhibition on ganglionic function in the SCG.^13^ Similar to previous reports,^31^ we found GAD65/67‐positive neurons in rat SCG slices (Figure 1A), and GABA_A_Rβ_2_ was expressed in both the cell body and axons of the SCG cultured neurons (Figure 1A). However, the SCG neurons showed an uneven distribution of GAD65/67, similar to Wolff, JR's results,^32^ indicating a feed‐forward inhibition system inside the SCG. Consistent with the inhibitory effect of GABA in the SCG, lentivirus knockdown of GABA_A_Rβ_2_ resulted in a significant increase in postganglionic activity, implicating the GABA_A_ receptor as a target. Diana Elinos et al^13^ suggested that GABA_A_Rα4 subunits are mainly expressed on principle ganglion neurons and that this subunit distribution is related to the level of GABA inhibition. In this study, we knocked down GABA_A_Rβ_2_ throughout the SCG without affecting the GABA_A_Rα4 subunit, and the data showed a thorough upregulation of sympathetic activity. Thus, we concluded that GABA_A_Rβ_2_ was the major target. Our data shown in Figure 1C, revealed that SR95531, the GABA_A_R antagonist binding αβγ‐composed receptors,^33^ only partially rescued intracellular Ca^2+^ downregulation by muscimol, indicating that the majority of these GABA_A_Rs are of the αβγ composition. Thus, we could not exclude the possibility that regional differences in GABA inhibition within the SCG were because of GABA_A_Rα4 subunit distribution or other subunits in the SCG.

**Figure 6:**
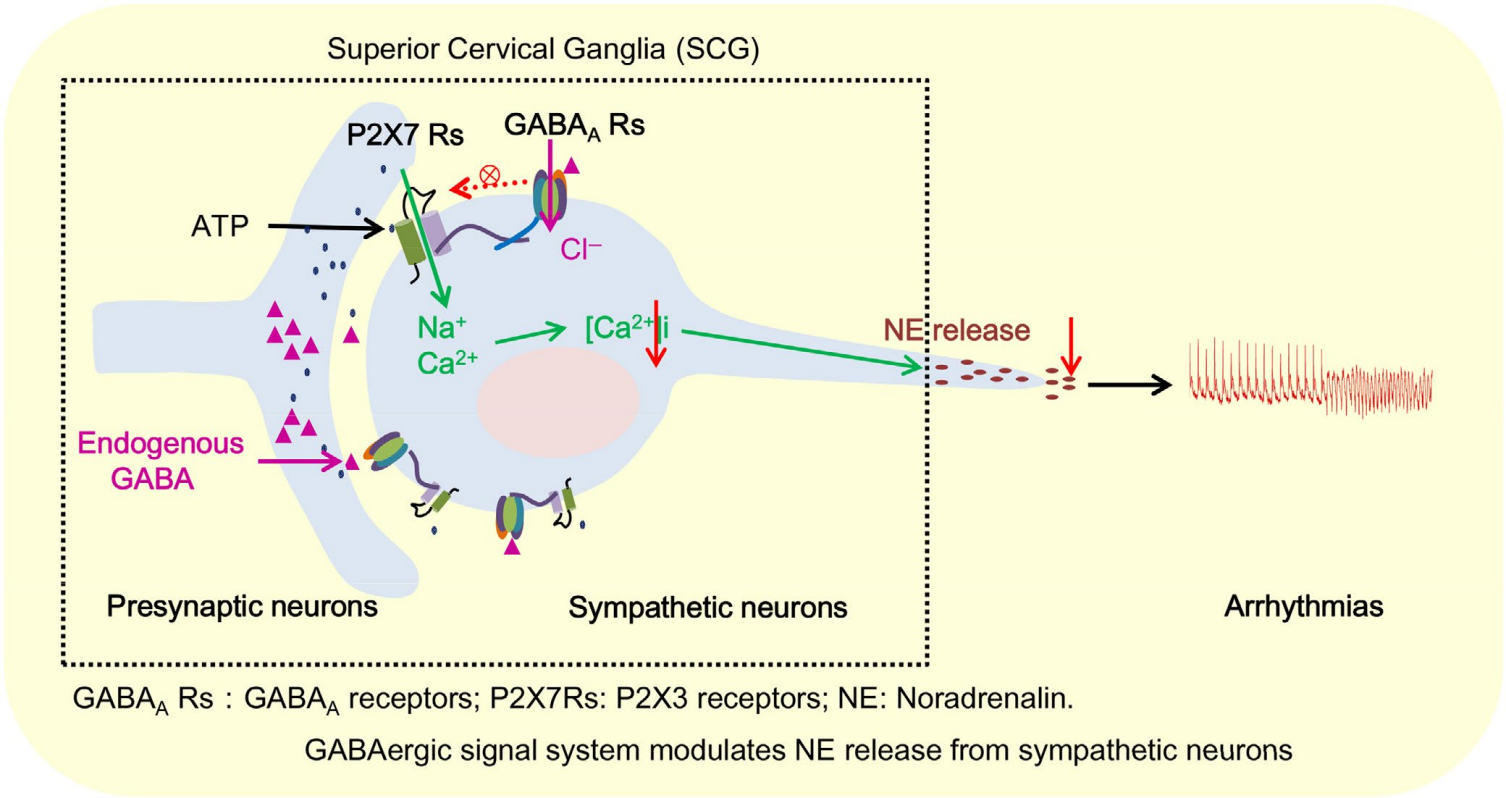
The schematic pathway of the SCG GABAergic signalling system involved in sympathetic activity and arrhythmias. SCG sympathetic neurons were positive for both P2X7Rs and GABA_A_Rs. GABA blocked the ATP‐induced [Ca^2+^]i elevation in vitro. Our results likely show another receptor‐receptor interaction model that is commonly observed in central neuronal synaptic functions. By inhibiting P2X7Rs, GABA blocks NE release from sympathetic neurons both inside and outside of cardiac tissues. By regulating sympathetic activity, GABA can attenuate arrhythmias in post‐MI rats, indicating that the GABAergic system could be a potential therapeutic target to protect the heart from arrhythmias post MI

### 3.2 GABA and NE release

NG Bowery and AL Hudson^34^ observed reduced [^3^H]‐noradrenaline secretion from sympathetic nerve endings in isolated atria in vitro, indicating a direct GABA inhibitory effect on the NE release of sympathetic nerve endings. Pharmacological tests revealed the dominant effect of GABA_A_R in NE release. As observed in our results, knockdown of SCG GABA_A_Rβ_2_ induced an elevation in plasma and cardiac NE levels and RSNA, thus increasing HR, which implied important roles of SCG GABA_A_R in downregulation of sympathetic nerve activity in vivo.

The mechanisms of GABA inhibition on NE release remain obscure. One hypothesis involves membrane hyperpolarization caused by GABA, thus leading to a decrease in NE output,^34^ but the underlying pathways require further investigation. Alternatively, NE secretion from nerve terminals is always dependent on intracellular Ca^2+^. Recent articles have provided clues showing that GABA may reduce ATP‐induced intracellular Ca^2+^ elevation through P2XRs in rat nodose ganglion neurons.^35^ A molecular study demonstrated an interaction of GABA_A_Rs and P2XRs in mouse spinal cord neurons and that GABA_A_R distribution and dynamics were modulated by P2XRs.^36^ Because SCG neurons were positive for both P2X7Rs and GABA_A_Rs, we examined changes in intracellular Ca^2+^ levels using two receptor agonists, ATP and muscimol. The reduction observed in the ATP‐induced intracellular Ca^2+^ elevation in isolated SCG neurons by muscimol in this study suggests the presence of inhibitory interactions between GABA_A_Rs and P2XRs for neuronal excitability.

### 3.3 GABAergic signalling system and arrhythmias

Post MI arrhythmias, especially VAs, are because of overactivation and sustained increases in cardiac sympathetic nerves in both clinic^37^ and basic^38^ research settings. Moreover, ameliorating the sympathetic nerve tone by RSN denervation could potentially reduce the incidence of VAs post MI.^39^, ^40^ In the present study, sympathetic tone was significantly increased post MI (Figure 3), and RSNA and NE levels were upregulated after SCG GABA_A_Rβ_2_ knockdown (Figure 4), suggesting that activation of SCG GABA_A_Rs reduces sympathetic tone to protect the heart from VA attack. In addition, after SCG GABA_A_Rβ_2_ knockdown, the survival rates were lowered to 30% or less from 70% in the sham surgery group, suggesting that the SCG GABAergic signalling system plays an important role. Technically, the knockdown was restricted to the SCG using a specific injection method; thus, the effect should be limited to inside and downstream of the SCG. Thus, targeting the SCG GABAergic signalling system may be a potential strategy to lower the incidence of VAs post MI.

Moreover, postganglionic fibres of the SCG participate in the formation of the cardiac plexus innervating myocardial tissues. The SCG transmits the sympathetic preganglionic signal and has an integration effect in the regulation of autonomic function.^41^ Additionally, the preganglionic nerves contain GABA varicosities.^13^ Moreover, in various medullary structures, GABA is involved in baroreflex function and sympathetic tone that can reduce the occurrence of arrhythmias.^42^ The blockade of CNS GABA_A_Rs in particular increases the sympathetic output to the heart.^18^ Imbalance in central GABA transmission has been associated with the development of cardiovascular diseases, including hypertension^43^ and heart failure.^44^ In addition, systemically administered GABA has been shown to reduce blood pressure in both spontaneously hypertensive rats and human subjects by inhibiting NE release from sympathetic nerve fibres and by decreasing peripheral sympathetic nerve activity.^45^, ^46^, ^47^ SCG GABA_A_Rβ_2_ knockdown might inhibit preganglionic function, thus leading to re‐evaluation in the brain. Our results showed that GABA partially reduced the inducible quotient of VAs and RSNA only, implying that other mechanism(s) in addition to the GABAergic signalling system are involved in VA progression. Following SCG GABA_A_Rβ_2 _knockdown, most rats died in the process of VA induction at day 7, indicating that SCG GABA_A_Rβ_2_ is very important in VAs. However, the underlying mechanisms require further investigation. Therefore, the central brain may have feasibly reduced sympathetic nerve activity partially through feedback mechanisms.

P2XR inhibition in the SCG reportedly depresses sympathoexcitation in a rat model of MI,^48^, ^49^ suggesting that P2XR blockage decreases sympathetic tone. Here, we demonstrated a physiological role of GABA_A_R inhibition in sympathetic nerve activity through P2X7Rs. However, we did not analyse the structural connections between GABA_A_Rs and P2X7Rs in the SCG, and these two receptors may work separately. According to previous reports,^35^, ^36^ we hypothesize (Figure 6) that GABA_A_Rs and P2X7Rs interact inside the cell membrane to exert an inhibitory function in SCG sympathetic neurons, but the mechanism requires further investigation.

In addition, a recent paper demonstrated that sympathetic denervation attenuated myocardial inflammation and improved cardiac function through the SCG post MI,^50^ which can somewhat explain our findings. In this regard, targeting the SCG GABAergic signalling system can protect the heart by inhibiting sympathetic nerve activity and cardiac inflammation post MI, although the detailed mechanisms need further exploration.

## 4 MATERIALS AND METHODS

The study was performed according to the protocols approved by the Institutional Animal Care and Use Committee of Qianfoshan Hospital and conformed to the US National Institutes of Health Guide for the Care and Use of Laboratory Animals.

### 4.1 Animals

Male Sprague‐Dawley rats (230 g‐280 g, 8 weeks old) provided by Vital River Laboratory (Beijing, China) were used in this study. All experimental procedures were performed in accordance with the institutional guidelines for animal care and research established by the Shandong University (Jinan, China) Institutional Animal Care and Use Committee.

During the experiment, all animals were housed in the SPF barrier system under a 12‐h light/dark cycle with the lights on beginning at 8 AM at a constant temperature of 22°C–24°C with free access to normal rat chow and drinking water. After experiments, all animals were killed by an overdose of pentobarbital sodium.

### 4.2 Experimental design

Protocol 1. For the exogenous GABA experiment, rats were randomized into the following three groups: group A, sham surgery; group B, MI; and group C, MI + exogenous GABA. GABA (A2129, Sigma‐Aldrich) was dissolved in water and intragastrically administered to animals at a dose of 500 mg/kg GABA in a volume of 500 μL^51^, ^52^ from one day before the experiment until the last day of the experiment. Rats in groups A and B were intragastrically administered water only. Seven days after ligation surgery, we performed experiments to collect hemodynamic measurements, electrophysiological data and renal sympathetic nerve activity (RSNA) to evaluate sympathetic nerve activity. At the end of the study, the SCG and heart tissue were collected and used for Western blotting, immunohistochemistry and ELISA.

Protocol 2. For the GABA_A_Rs β_2_ subunit (GABA_A_Rβ_2_) knockdown experiment, rats were randomly divided into the following four groups: sham surgery + vehicle; sham surgery + GABA_A_Rβ_2_ shRNA lentiviral vector; MI + vehicle; and MI + GABA_A_Rβ_2_ shRNA lentiviral vector. Vehicle and GABA_A_Rβ_2_ shRNA lentiviral vectors were delivered by targeted microinjection into the SCG four days before MI surgery. Before death on day 7 post MI, electrophysiological testing and RSNA were performed. Then, heart tissues, blood samples and the SCG were collected for Western blotting, qRT‐PCR, immunohistochemistry and ELISA.

Protocol 3. For the experiment performed to determine the underlying molecular mechanism of GABA involved in the reduction in peripheral sympathetic activity, sympathetic neurons isolated from the foetal SCG were used. In this protocol, Ca^2+^ imaging and immunofluorescence were performed.

### 4.3 Preparation of the lentiviral GABA_A_Rβ_2_‐siRNA vector

Three selected lentiviral siRNAs targeting different sites of the rat GABA_A_Rβ_2_ gene were designed and constructed by GENE company (China). Green fluorescent protein (GFP) expression was used as a marker for transduction efficiency.

GABA_A_Rβ_2 _Sequence 1: 5ʹ‐CCGGCACCTACTTCCTGAATGATAACTCGAGTTATCATTCAGGAAGTAGGTGTTTTT‐3ʹ;

Sequence 2: 5ʹ‐CCGGAAGCAGCTGAGAAAGCTGCTACTCGAGTAGCAGCTTTCTCAGCTGCTT TTTTT‐3ʹ;

Sequence 3: 5ʹ‐CCGGGAGCTTCCTCAGTTCTCCATTCTCGAGAATGGAGAACTGAGGAAGCTCTTTTT‐3ʹ.

The shRNA sequence targeting the enhanced GFP (eGFP) reporter gene (sequence: 5ʹ‐CACCGTTCTCCGAACGTGTCACGTCAAGAGATTACGTGACACGTTCGGAGAATTTTTTG‐3ʹ) was included as a control. Viral titres were determined using qPCR.^53^ The lentivirus GABA_A_Rβ_2_‐shRNA titre was 8 × 10^8^ vector genomes (Tu/mL), and the lentivirus GFP titre was 8 × 10^8^ Tu/mL.

### 4.4 SCG microinjection

After rats were anaesthetized with 3% pentobarbital sodium (30 mg/kg ip), we performed a 2‐centimetre vertical neck incision. The salivary glands were then exposed, and the underlying muscles were lifted and moved aside to expose the carotid triangles and carotid bifurcation. The almond‐shaped SCG was located behind the carotid bifurcation. The shRNA lentiviral vector was then delivered to the SCG using a microinjector.

### 4.5 Animal model

After rats were anaesthetized with 3% pentobarbital sodium (30 mg/kg ip), intubated via tracheotomy, and ventilated with a small‐animal ventilator, a 3‐centimetre left thoracotomy was performed through the left fourth intercostal space. Then, the heart was exposed through pericardiotomy. The left anterior descending coronary artery was ligated 2 mm from its origin between the pulmonary artery conus and left atrium to induce MI as previously described with 6/0 silk sutures.^54^ During this experiment, an animal biological function experiment system (BL‐420S, TaiMeng, China) was used to monitor the electrocardiogram (ECG). MI was confirmed by ST elevation on a surface ECG and stiff movement of the left ventricle (LV). The sham group underwent thoracotomy and pericardiotomy only. After the surgery, rats were allowed to recover from the anaesthesia on a heated pad to maintain a body temperature of 37°C and then returned to their individual cages.

### 4.6 Quantitative real‐time PCR

Total RNA was extracted from the SCG with the EZNA Total RNA Kit II (OMEGA Biotek, Inc, Norcross, GA, USA) following the manufacturer's protocol. cDNA was synthesized using a Prime Script RT Reagent Kit (Thermo Fisher, Grand Island, NY, USA). qRT‐PCR was performed with a ViiA7 Real‐Time PCR System (Applied Biosystems, Waltham, MA, USA). Relative expression was calculated using GAPDH as an endogenous internal control. Relative gene expression was calculated by the 2‐ΔΔCT method^55^ and normalized to GAPDH expression. The primers used in this study were as follows:

GABA_A_Rβ_2 _primers: Forward, 5′‐CCCTGTGGCAGTAGGAATGAA‐3′; reverse, 5′‐GCCACTCGATTGTCCAAAGTC‐3′.

GAPDH primers: Forward, 5′‐AGATCCACAACGGATACATT‐3′; reverse, 5′‐TCCCTCAAGATTGTCAGCAA‐3′.

### 4.7 SCG neuron culture

The foetal rat SCG dissection protocol has been described elsewhere.^56^ Female rats at the desired gestational stage of 18‐20 days were anaesthetized with 3% pentobarbital sodium, embryos were removed and decapitated, and the heads were placed in precooled fresh culture medium containing 10% foetal calf serum. SCGs were isolated using a stereoscopic microscope and were enzymatically digested with prewarmed 0.125% trypsin for 15 minutes. After gentle mechanical disruption with a Pasteur pipette, cells were transferred into petri dishes maintained in Neurobasal Medium with B27 (A3582801, Gibco) serum‐free supplement and Glutamax‐1. The cells were incubated at 37°C in a 10% CO_2_ humidified atmosphere.

### 4.8 Ca^2+^ imaging

Cultured SCG neurons on day 7 were used to perform Ca^2+^ imaging. Neurons were washed with Hank's balanced salt solution without Ca^2+^ and Mg^2+^ three times every five minutes and then covered with a sufficient amount of Fura‐Red AM (10 μm, F3021, Invitrogen) with PluronicF‐127 (P6867, Invitrogen) in Hank's balanced salt solution without Ca^2+^ and Mg^2+^ at 37°C for at least 60 minutes. Cells were incubated for another 30 minutes with Hank's balanced salt solution with Ca^2+^ and Mg^2+^. Images were immediately stored on a high‐speed hard drive. The ratio of fluorescence intensity changes excited at 488 nm and collected at 625‐725 nm was computed as previously reported.^57^, ^58^, ^59^ The formula for calculating fluorescence intensity changes is ΔF/F = (F‐Fbase)/(Fbase‐B), where F indicates the real fluorescence intensity, Fbase is the intracellular fluorescence intensity before stimuli, and B is the background fluorescence intensity. Then, ATP (10 µM, A1852, Sigma‐Aldrich), muscimol (10 µM, G019, Sigma‐Aldrich) or (and) SR95531 (1 µM, S106, Sigma‐Aldrich) were added to the extracellular fluid to measure the ratio of fluorescence intensity at 488 nm. Pictures were taken every 1 minute.

### 4.9 Western blotting

Equal amounts of SCG protein samples were separated on 8%‐12% SDS‐PAGE gels and transferred onto polyvinylidene difluoride membranes. Then, the membranes were blocked with 5% fat‐free milk in TBS containing 0.05% Tween 20 for 2 hours. Membranes were incubated overnight at 4°C with primary antibodies against GABA_A_Rβ_2_ (1:1000; Abcam) and GAPDH (1:5000; CoWin Bioscience, Beijing, China). After the membranes were washed thrice with TBST, they were incubated with the corresponding secondary antibodies (1:5000). The bands were visualized using an enhanced chemiluminescence kit (Millipore, Billerica, MA, USA) with a FluorChem E Imager (Protein‐Simple, Santa Clara, CA, USA).

### 4.10 RSNA recording

The left RSNA was measured as previously described.^60^ The animals underwent a retroperitoneal incision, and a bundle of the left renal sympathetic nerve was carefully dissected from the other connective tissues for the RSNA recording preparation. The nerve was cut distally to eliminate its afferent activity. The central end of the nerve was placed on a pair of platinum electrodes. To insulate the electrodes and the nerve from the surrounding tissue and to prevent desiccation of the nerve, we covered the electrodes and the nerve in warm mineral oil. The signals were amplified, filtered and monitored with Lab‐Chart Pro software (AD Instruments, Sydney, Australia) with a high‐pass filter at 10 Hz and a low‐pass filter at 3000 Hz. The RNSA was recorded at a time constant of 100 ms The background noise was measured after sectioning the central end of the nerve at the end of each experiment, and the experimental data were corrected for this value.

### 4.11 Electrophysiological study

Rats were reanaesthetized and mechanically ventilated as described above. The protocol used for the programmed electrical stimulation was performed as previously reported.^54^, ^61^ After monitoring the surface ECG, a programmed stimulation protocol was performed using electrodes implanted on the epicardial surface of the LV. Programmed electrical stimulation was performed to induce VAs as follows: eight paced beats at a cycle length of 120 ms (S1) were applied, followed by one to three extrastimuli (S2, S3 and S4) at shorter coupling intervals. The pacing protocols were interrupted if ventricular tachycardia was induced. Ventricular tachyarrhythmias, including ventricular tachycardia and ventricular fibrillation, were considered nonsustained when they lasted <15 beats and sustained when they lasted >15 beats. The VA scores were determined by the inducibility quotient of the ventricular tachyarrhythmias as follows: 0, noninducible; 1, nonsustained tachyarrhythmias induced with three extrastimuli; 2, sustained tachyarrhythmias induced with three extrastimuli; 3, nonsustained tachyarrhythmias induced with two extrastimuli; 4, sustained tachyarrhythmias induced with two extrastimuli; 5, nonsustained tachyarrhythmias induced with one extrastimulus; 6, sustained tachyarrhythmias induced with one extrastimulus; 7, tachyarrhythmias induced during a train of eight stimuli (8 × S1) at a basic cycle length of 120 ms; and 8, heart stopped before programmed electrical stimulation. When multiple forms of arrhythmias occurred in one heart, the highest score was used.^62^ The experimental protocols were typically completed within 10 minutes.

### 4.12 Hemodynamic measurements

A 2F microtip pressure‐volume catheter (SPR‐869, Millar, Houston, TX, USA) was inserted into the LV from the right carotid artery to obtain the pressure‐volume (P‐V) data. The Lab‐Chart Pro software (AD Instruments, Australia) was used to evaluate the LV end‐systolic pressure (LVESP), LV end‐diastolic pressure (LVEDP), maximal slope of the LV systolic pressure increment (dp/dtmax), diastolic pressure decrement (dp/dtmin), and LV ejection fraction (EF). At the end of the experiment, the volume was calibrated using the relative volume unit (RVU) calibration method with fresh heparinized warm blood as previously reported.^61^

### 4.13 Immunohistochemistry and Masson's trichrome staining

For Masson's trichrome staining, hearts were harvested from each group and sliced into 3 portions along the top and bottom edges of the infarction. The middle portion included the whole infarcted myocardium and was immersed in 10% formalin for 24 hours, embedded in paraffin, cut into 10‐μm‐thick sections and stained with Masson's trichrome. The infarct size (%) was expressed as the mean percentage of the infarcted LV versus the inner and outer LV circumferences. To ensure the clinical importance of the data, only rats with a moderate infarct size (30%‐50%) were enrolled.^62^

For immunofluorescence, cultured SCG neurons were prepared according to a standardized process and then incubated with anti‐tyrosine hydroxylase (TH) Ab (1:400; Millipore), anti‐GABA_A_Rβ_2_ Ab (1:200; Abcam) and anti‐P2X7 Ab (1:150, Abcam) overnight at 4°C. After three washes with PBS, neurons were incubated with FITC‐conjugated rabbit anti‐sheep (1:200; Bethyl Laboratories, Montgomery, TX) and Alexa 545‐conjugated goat anti‐rabbit (1:100; Peprotek) secondary antibodies. The sections were counterstained with DAPI (Life Technologies, Grand Island, NY) to identify nuclei.

### 4.14 ELISA

Blood and cardiac tissue samples were collected for norepinephrine (NE) determination on day 7 after MI surgery. The blood was placed into Eppendorf tubes containing 10% EDTA‐Na2 (30 µL, 1 mg/mL, Sigma‐Aldrich) and 40 µL aprotinin (500 kIU/mL, Sigma‐Aldrich). Myocardia were minced and suspended in 0.4 N perchloric acid with 5 mmol/L reduced glutathione (pH 7.4). After immediate centrifugation, the supernatants were collected and stored at −80°C. We used a commercial NE ELISA kit (USCN Life Science, Wuhan, China) to measure the NE concentration.

### 4.15 Statistical analysis

Statistical analysis was performed with SPSS 17.0. Quantitative data are presented as the mean ± SE. Differences among groups were analysed by t test or ANOVA followed by Least Significant Difference testing. A value of *P* < 0.05 was considered significant.

## 5 CONCLUSIONS

The SCG GABAergic signalling system plays a key role in mediating peripheral sympathoexcitation post MI via inhibition of intracellular Ca^2+^ concentrations in sympathetic neurons, which might be a new mechanism responsible for VAs post MI.

## CONFLICTS OF INTEREST

There are no conflicts of interest.
