# Supplementary figures and images for: A novel sympathetic neuronal GABAergic signalling system regulates NE release to prevent ventricular arrhythmias after acute myocardial infarction
Source: Acta Physiol (Oxf). 2019 Jun 12;227(2):e13315. doi: 10.1111/apha.13315 (PMC6813916; doi:10.1111/apha.13315)

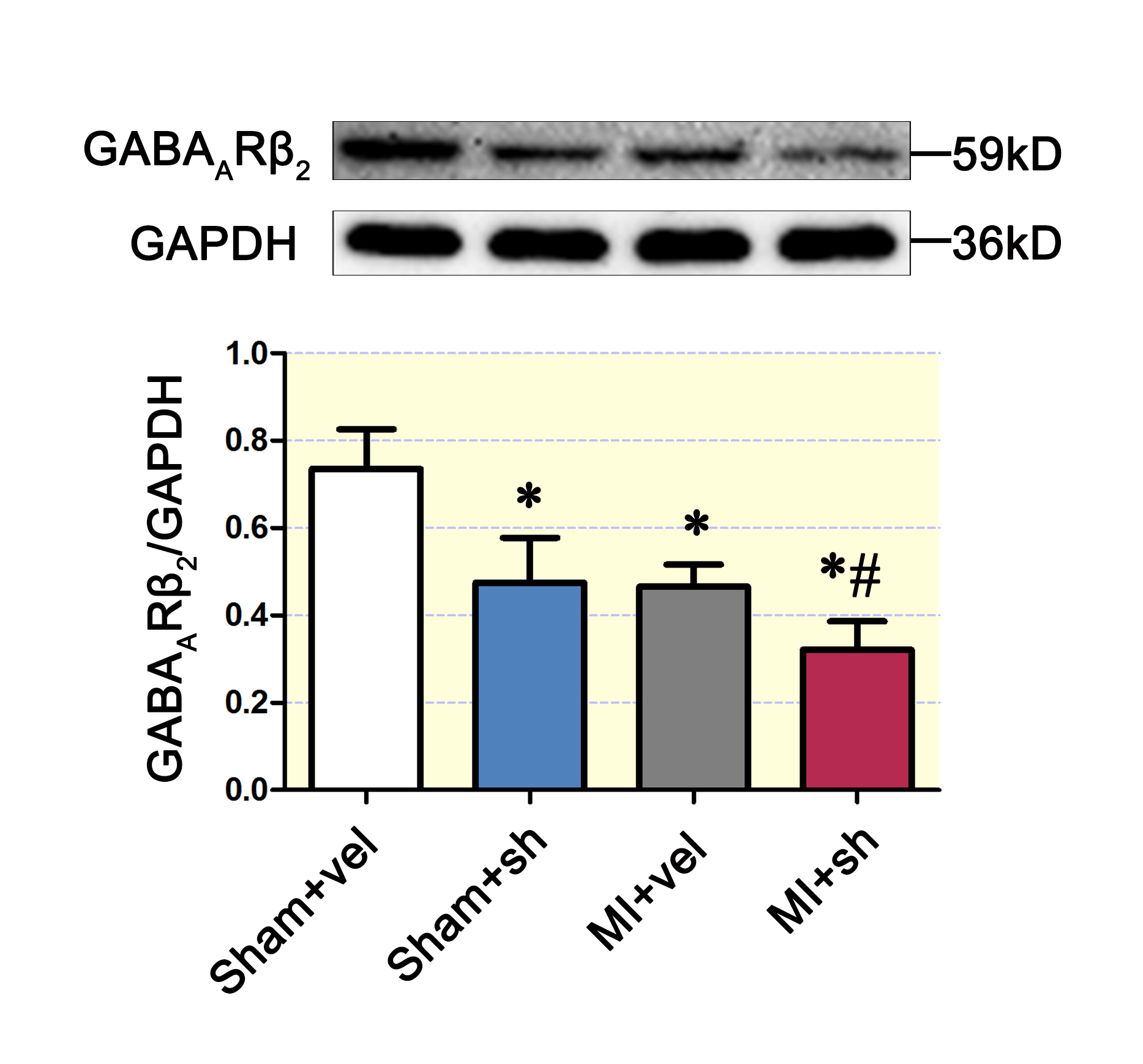

Supplement: Supplementary file 1 [file APHA-227-na-s001.jpg]
